# Alzheimer’s Disease and Epilepsy: Exploring Shared Pathways and Promising Biomarkers for Future Treatments

**DOI:** 10.3390/jcm13133879

**Published:** 2024-07-01

**Authors:** Athanasios-Christos Kalyvas, Maria Dimitriou, Panagiotis Ioannidis, Nikolaos Grigoriadis, Theodora Afrantou

**Affiliations:** 2nd Department of Neurology, AHEPA University Hospital, Aristotle University of Thessaloniki, GR54636 Thessaloniki, Greece; kalthaxr@hotmail.com (A.-C.K.); dimitrioumariad@gmail.com (M.D.); ioannidispanosgr@yahoo.gr (P.I.); grigoria@med.auth.gr (N.G.)

**Keywords:** Alzheimer’s disease, epilepsy, late-onset epilepsy of unknown etiology, LOEU, pathogenetic mechanisms, anti-epileptic drugs, biomarkers

## Abstract

**Background**: Alzheimer’s disease (AD) and epilepsy represent two complex neurological disorders with distinct clinical manifestations, yet recent research has highlighted their intricate interplay. This review examines the association between AD and epilepsy, with particular emphasis on late-onset epilepsy of unknown etiology, increasingly acknowledged as a prodrome of AD. It delves into epidemiology, pathogenic mechanisms, clinical features, diagnostic characteristics, treatment strategies, and emerging biomarkers to provide a comprehensive understanding of this relationship. **Methods**: A comprehensive literature search was conducted, identifying 128 relevant articles published between 2018 and 2024. **Results**: Findings underscore a bidirectional relationship between AD and epilepsy, indicating shared pathogenic pathways that extend beyond traditional amyloid-beta and Tau protein pathology. These pathways encompass neuroinflammation, synaptic dysfunction, structural and network alterations, as well as molecular mechanisms. Notably, epileptic activity in AD patients may exacerbate cognitive decline, necessitating prompt detection and treatment. Novel biomarkers, such as subclinical epileptiform activity detected via advanced electroencephalographic techniques, offer promise for early diagnosis and targeted interventions. Furthermore, emerging therapeutic approaches targeting shared pathogenic mechanisms hold potential for disease modification in both AD and epilepsy. **Conclusions**: This review highlights the importance of understanding the relationship between AD and epilepsy, providing insights into future research directions. Clinical data and diagnostic methods are also reviewed, enabling clinicians to implement more effective treatment strategies.

## 1. Introduction

Dementia poses a significant global health problem, affecting over 55 million people worldwide, according to the World Health Organization [1]. Alzheimer’s disease (AD) carries the highest burden among all types of dementia, accounting for 60–70% of dementia cases [1]. The prevalence of AD increases with age, rising from 10% in people aged ≥65 years to 32% in those aged ≥85 years [2]. By 2050, the number of people with AD is projected to triple to 152 million due to the aging population [2]. Clinically, AD patients primarily experience cognitive decline, particularly in memory, along with mood, behavior, and daily activity impairments. Mild cognitive impairment (MCI), characterized by subtle memory, executive function, or visuospatial problems that do not significantly interfere with daily life, often precedes the onset of AD and significantly increases the risk of future AD diagnosis [2].

Simultaneously, epilepsy affects 50 million people worldwide. The incidence of epilepsy peaks in childhood and again after the age of 65. Among individuals over 85 years old, the incidence of epilepsy reaches 180 cases per 100,000 population [3,4]. Late-onset epilepsy (LOE) is defined as the occurrence of epilepsy in individuals older than 60 years of age [5], however the cut off age ranges from 55 to 67 years in different studies [6]. In this group, 25–50% of epilepsy cases lack a recognizable cause, termed late-onset epilepsy of unknown etiology (LOEU) [7]. Similar to AD, the aging population is expected to increase the incidence of epilepsy and cases of LOEU [8].

Recent research indicates a reciprocal relationship between AD and epilepsy, particularly LOEU [4,9] with each condition increasing the risk for the other [4,6,8,10]. Epidemiological data, combined with the discovery of shared pathogenetic mechanisms, provide a robust rationale for this relationship. Furthermore, biochemical, imaging, and neurophysiological findings, alongside insights into the use of anti-seizure medications, emerging biomarkers, and animal studies, underscore the importance of thoroughly investigating the connection between AD and epilepsy. Such research holds great promise for enhancing clinical management strategies. This review compiles relevant data, with a particular emphasis on current knowledge regarding the pathogenetic mechanisms involved, aiming to serve as a comprehensive guide, that increases clinicians’ awareness and informs appropriate treatment strategies for these patients.

## 2. Materials and Methods

**Figure 1:**
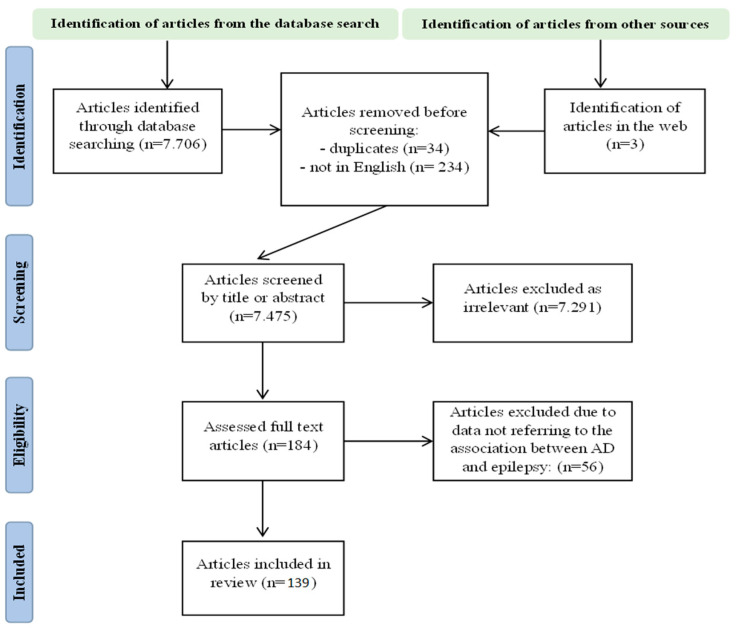
Flowchart for a review of the association between Alzheimer’s disease and epilepsy. AD: Alzheimer’s disease.

A comprehensive literature search was conducted across PubMed, Medline, and Scopus databases using the terms “Alzheimer”, “dementia”, “AD”, “epilepsy”, “seizures”, “epileptic”, and “epileptiform” in titles and abstracts. The search spanned from 2018 to 2024 aiming to identify articles examining the association between AD and epilepsy. The included studies addressed various aspects, including epidemiology, speculated pathogenetic mechanisms, clinical features, diagnostic characteristics, treatment strategies, findings from animal studies, and biomarkers. Only articles written in English were considered. After screening by title and abstract, relevant articles were reviewed in full text. Exclusions included articles in languages other than English, as well as those addressing disorders unrelated to AD or epilepsy. Duplicate articles were also removed. Ultimately, 128 articles met the criteria for inclusion in this review. The Prisma flowchart is shown in Figure 1.

## 3. Results

### 3.1. Epidemiological Data Describing the Comorbidity of Alzheimer’s Disease and Epilepsy

Initially, it was believed that epilepsy was a late complication of AD [11], with seizures appearing 4–6 years after the diagnosis of AD [12]. However, it is now increasingly recognized that epilepsy may be present from the onset of cognitive decline or even precede it [4,13,14]. Recent studies indicate that 10–22% of AD patients may experience at least one epileptic seizure [13], with rates as high as 64% reported in some studies [7]. Moreover, two-thirds of these patients will experience additional episodes within 24 h, without an evident etiological factor [15].

A systematic review by Dun et al. found that AD elevates the risk for epilepsy threefold [16], whereas epilepsy was 17 times more prevalent among AD patients compared to the general population [17]. According to Vossel et al., 83% of AD/MCI patients had epilepsy prior to or at the time of diagnosis, with seizure onset occurring simultaneously with or before cognitive deterioration in 38% of them [7]. Additionally, studies observed this trend in MCI diagnosis, particularly amnestic MCI (aMCI), with epilepsy onset occurring 4.5 years before AD and 2.7 years before MCI [7]. Patients with LOEU exhibited an onset of dementia due to AD approximately six years earlier than non-epileptic adults [18].

A distinct form of AD, known as autosomal dominant or early onset AD (occurring before age 65), is characterized by genetic predisposition, a more severe phenotype, and earlier cognitive decline onset. Individuals with this form of AD also have a significantly higher risk for epilepsy [11,13,19,20]. Among carriers of presenilin 1 (*PSEN1*), presenilin 2 (*PSEN2*), amyloid precursor protein (*APP*) mutations, or *APP* duplications with AD, as many as 47.7% experienced seizures during an 8.4-year follow-up period [13,21]. In other words, AD patients aged 50–59 had an 87-fold increased risk of seizures compared to age-matched individuals without AD [13]. Additionally, patients carrying these mutations have a 28% higher risk of seizures compared to those with sporadic AD [22]. In this group of patients, 3–7% had seizures preceding AD diagnosis [7,23]. This predisposition to seizures is attributed to early amyloid β pathology, which damages synaptic neurotransmission and leads to neuronal hyperactivity [23,24].

Conversely, epilepsy is associated with a 2- to more than 3-fold increase in the risk of dementia and AD [4,6,8,12,15,16]. Approximately 0.6% to 17.5% of epilepsy patients have dementia, most commonly AD [9,12,17], with rates reaching up to 35% in certain patient groups [25]. Cognitive deficits are detected in up to 80% of epilepsy patients [12]. Regarding LOE, 42% of affected individuals eventually develop dementia [6] with 22% experiencing dementia within the first 10 years after LOE onset, particularly among those with MCI or low cerebrospinal fluid (CSF) amyloid-beta (Aβ) 42 levels reflecting a three-fold increased risk [26,27]. A 5-year follow-up of LOEU patients revealed that 21–25% developed overt dementia with 17.5% being diagnosed with AD [6], emphasizing the need for vigilance regarding imminent cognitive deterioration in individuals with LOEU [7] while also monitoring epileptic activity, as increased activity correlates with AD worsening [8].

Epilepsy not only acts as a risk factor but also adversely affects cognition in AD patients. The rate of decline in Mini-Mental State Examination (MMSE) scores was faster in AD patients with epileptiform activity and in epileptic patients [20,26]. Conversely, treatment with anticonvulsant agents yielded favorable cognitive outcomes [26]. MCI patients with epilepsy demonstrated poorer cognitive performance compared to those without epilepsy [26]. LOE is associated with poorer cognitive function, as assessed by measures such as MMSE, dementia rating scale, and Montreal cognitive assessment. Specifically, LOE impacts domains including verbal and visual memory, executive function, language, psychomotor skills, and processing speed [9]. It is important to note that LOE patients with dementia are less likely to achieve seizure freedom for 12 months or more and demonstrate poorer responses to anti-seizure medication [25]. Electroencephalographic markers such as subclinical epileptiform activity (SEA) in AD patients were linked to earlier cognitive deterioration onset [28], lower scores in several test batteries, and faster cognitive decline during follow-up [29,30,31] whereas left temporal spikes and increased spike frequency correlated with more pronounced cognitive decline [30]. Furthermore, SEA appears to interact with sleep in AD patients, reducing rapid eye movement (REM) sleep and causing sleep-disordered breathing [32]. One hypothesis proposes that accelerated tau protein (tau) accumulation resulting from seizures or a more aggressive form of AD may explain the increased cognitive decline observed in epileptic AD patients [33].

### 3.2. Risk Factors Implicated in Both Alzheimer’s Disease and Epilepsy

#### 3.2.1. Shared Risk Factors between Alzheimer’s Disease and Epilepsy

It is evident that AD and epilepsy share many common risk factors, which may partly explain the interaction between these two conditions (Table 1. Common risk factors for Alzheimer’s disease and epilepsy). Genetic factors, such as mutations in the *PSEN1*, *PSEN2*, and *APP* genes along with *APP* duplications, are associated with increased Aβ production. This genetic predisposition results in early-onset AD (<65 years old) with a worse prognosis compared to sporadic AD. Notably, Aβ accumulation contributes to heightened neuronal excitability and an increased risk of seizures [28,34].

The apolipoprotein ε4 allele (*APOEε4*) is a primary genetic risk factor for sporadic AD and is also implicated in LOE. The risk of LOE increases with the number of ε4 alleles present. Apolipoprotein E (APOE) plays a critical role in synaptic protein expression, neuronal differentiation, and cholesterol metabolism. Microglia and astrocytes carrying the *APOEε4* allele exhibit reduced capacity for Aβ uptake [4,7]. In addition, *APOEε4* may influence neuronal excitability through its effects on inflammation, cerebrovascular integrity, and overall homeostatic mechanisms. In regions such as the hippocampus and cortex, APOEε4 can alter structural and functional aspects of neuronal cells, disrupting inhibitory network function [39]. Electroencephalogram (EEG) studies on first-degree relatives of AD patients and asymptomatic APOEε4 carriers have shown high-voltage slow waves and sharp waves during hyperventilation, indicating a higher risk of epilepsy in individuals at risk of AD [40].

The triggering receptor expressed on the myeloid cells 2 (*TREM2*) gene may also connect AD and epilepsy. The *R47H* variant of the *TREM2* gene, associated with a 2–4-fold increased risk of AD, has been found to elevate epileptic activity in mouse models [35] Genetic studies have identified multiple genes, including *APOE*, that are implicated in common pathogenetic mechanisms between AD and epilepsy. These genes include *CLU*, *TNFRSF21*, *MS4A*, *ABCA7*, *HMHA1*, *MARK4*, *BIN1*, and *APOC1* [36].

Regarding vascular risk factors, Cretin et al. concluded that epilepsy in AD patients might result from a combination of amyloid and small vessel pathology [41]. Vascular risk factors contribute to both AD and LOE [42] and constitute modifiable risk factors for both dementia and epilepsy [43]. Dyslipidemia, diabetes, and hypertension increase the risk of stroke, which is the most common cause of LOE [38]. More than 50% of LOE patients with cerebral small vessel disease have deficits in verbal memory, processing speed, attention, executive functions, and visuospatial perception [44]. Therefore, screening for vascular abnormalities in both AD and epileptic patients is imperative. However, a study adjusting for vascular risk factors suggested that other factors might also contribute to the increased risk of dementia following LOE [45].

Blood–brain barrier (BBB) dysfunction is speculated to contribute to AD/MCI pathogenesis and is also considered a critical mechanism in epilepsy, where it is associated with albumin extravasation. BBB dysfunction is linked to several pro-epileptic effects, including impaired astrocyte function, neuroinflammation, synaptic dysfunction leading to impaired plasticity and excitatory synaptogenesis, hyperexcitability, and alterations in extracellular structures. In AD patients, BBB dysfunction has been associated with paroxysmal slow wave events, which are correlated with the presence of albumin resulting from BBB dysfunction [46].

A history of traumatic brain injury is recognized as a risk factor for AD and has also been associated with epilepsy. This association may be attributed to increased tau expression and the formation of phosphorylated Tau following the injury [47,48].

#### 3.2.2. Risk Factors for Epilepsy in Individuals with Alzheimer’s Disease

Several factors increase the risk of epilepsy in individuals with AD. These include early-onset AD, male sex, older age, disease duration, severity, imaging abnormalities affecting the precuneus and parietal lobe atrophy, and treatments affecting seizure threshold, may contribute. Furthermore, cerebrovascular abnormalities and comorbidities such as hypertension, diabetes and dyslipidemia as well as traumatic brain injury further heighten the likelihood of epilepsy [27,49]. Myoclonus has been reported as a risk factor for seizures in AD [50]. Moreover, higher education level, specific cognitive test scores, and higher CSF total tau protein (tTau) levels have been identified as additional risk factors for epilepsy in AD [51,52].

Patients with dementia who develop epilepsy experience a higher mortality rate, which is mostly attributed to comorbidities. However, in 18% of cases, factors associated with epilepsy are identified as contributing to mortality [38].

#### 3.2.3. Risk Factors for Dementia in Epileptic Patients

Advanced age or advanced age at epilepsy onset (over 60 years old) increases the likelihood of (suspected) dementia diagnosis by 6.1 and 2.9 times, respectively [6]. Cardiovascular parameters also serve as additional risk factors for dementia in the course of epilepsy [53]. In epileptic patients, factors such as male sex, lower education level, seizure frequency, severity, duration, focal to generalized tonic-clonic seizures, and depression are suspected risk factors for dementia [9,54,55]. Additionally, consideration of the cognitive effects of antiepileptic drugs is crucial [9].

### 3.3. Shared Pathogenetic Mechanisms between Alzheimer’s Disease and Epilepsy

**Figure 2:**
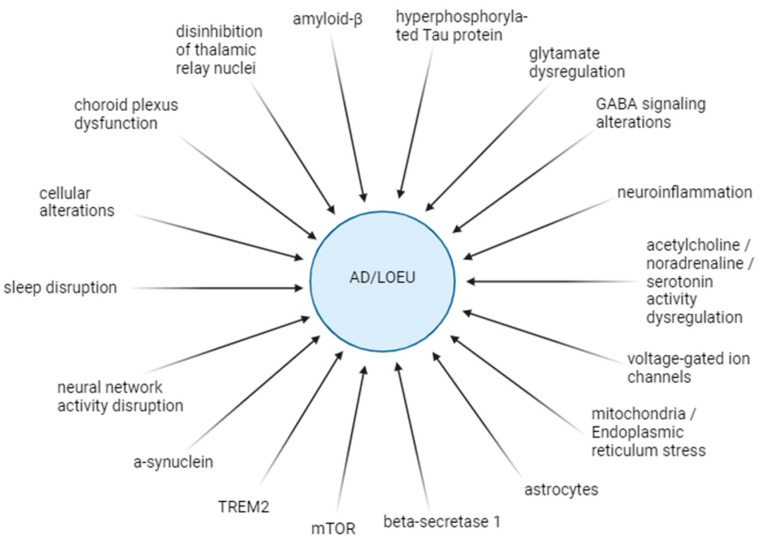
Pathogenetic mechanisms involved in Alzheimer’s disease and late-onset epilepsy of unknown etiology. GABA: gamma-aminobutyric acid; mTOR: Mechanistic target of rapamycin; TREM2: Triggering receptor expressed on myeloid cells 2.

The robust association between AD and epilepsy underscores the importance of investigating their shared pathogenetic mechanisms, which is a current research priority. The pathological hallmark of AD includes the extracellular aggregation of Aβ and the formation of neurofibrillary tangles composed of hyperphosphorylated tau (pTau) protein intracellularly. These changes predominantly affect the cortex and limbic areas such as the temporal lobe and hippocampus [4,56]. These pathological changes eventually result in synapse degeneration and neuronal loss, accompanied by neuroinflammation. Moreover, the cerebrovascular, cytoskeletal, and structural alterations observed in AD may also contribute to the development of epilepsy [13]. Epileptic seizures, in turn, have been shown to increase the deposition of Aβ and pTau, thereby exacerbating the progression of AD pathology and cognitive decline [4,57]. Additional common findings in both AD and epilepsy include temporal lobe atrophy and glial cell hyperplasia [12]. These changes often result in the dysregulation of the balance between excitatory and inhibitory activity, leading to elevated network hyperexcitability, a shared characteristic observed in both conditions [56,58]. Figure 2 illustrates the pathogenetic mechanisms involved in the association between AD and LOEU.

#### 3.3.1. Amyloid-β

While Aβ pathology is considered age-dependent and not directly linked to cognitive decline in epileptic patients [59], it is prominent from a young age in early-onset AD patients, who face a heightened risk of seizures. In a mouse model, hyperexcitable neurons were found to surround amyloid plaques, and epileptic activity correlated positively with plaque accumulation [60]. However, emerging evidence suggests that it is not the amyloid plaques themselves but rather the oligomeric form of Aβ that plays a central role in epileptogenicity in AD [61].

The interaction between Aβ oligomers and voltage-dependent channels is believed to trigger Ca^2+^ influx into neurons, resulting in short-term glutamate release [4,50]. Consequently, increased glutamate release and hindered reuptake from astrocytes lead to elevated glutamate concentration, which stimulates N-methyl-D-aspartate (NMDA) receptor signaling. This procedure promotes neuronal excitability fostering hypersynchronization and, ultimately, epilepsy [4,12,58,62]. Additionally, it induces cell death and cognitive decline [8,63]. Memantine exerts its beneficial effects on cognition by reducing Ca^2+^ influx [4]. However, over the long term, Aβ promotes NMDA receptor endocytosis but at that time regression may not be feasible [50]. Furthermore, elevated glutamate levels trigger the release of proinflammatory cytokines locally [62]. Aβ impairs calcium homeostasis in both neurons and glial cells, leading to structural alterations in neurons and anomalies in neurotransmission [21].

This scenario is often accompanied by the inhibition of sodium channels in inhibitory interneurons, thereby affecting seizure threshold [21,22,63] by reducing gamma-aminobutyric acid (GABA) signaling [27]. Potassium signaling may also be particularly impeded [21]. Additionally, Aβ-mediated upregulation of glycogen synthase kinase-3 beta (GSK3β) activity has been observed [64]. Aβ induces detrimental synaptic effects, including damage, alterations in plasticity, and impaired coordinated network activity, particularly in the hippocampus [56,65]. Recent research suggests that the D1 receptor may serve as a mediator of the epileptogenicity of Aβ42, leading to the rearrangement of α-amino-3-hydroxy-5-methyl-4-isoxazole propionic acid (AMPA) receptor units after the presence of Aβ42 oligomers [66].

Once Aβ induces epilepsy, epileptic activity further promotes the formation and accumulation of Aβ and pTau [62]. Aβ pathology begins more than 20 years before the onset of cognitive symptoms and precedes tau protein pathology. Despite initially inducing hyperexcitability, Aβ ultimately leads to decreased brain activity [67]. Reductions in Aβ and *APP* levels have been shown to decrease excitability in mouse models [7,60], though they did not delay cognitive impairment. These observations suggest the involvement of additional mechanisms [48].

#### 3.3.2. Hyperphosphorylated Tau Protein

Epileptic patients, even in the absence of AD, exhibit a higher burden of pTau pathological changes compared to healthy individuals. This elevation in pTau levels correlates with the frequency of seizures and cognitive decline, emphasizing the significant role of pTau in epilepsy and its involvement as a tauopathy [4,59,68]. The increased presence of pTau in epilepsy is distinct in patients with a recognized cause of epilepsy and in patients with LOEU, where it is more pronounced. This suggests that it is not the seizure characteristics themselves that increase pTau, but rather the underlying etiological factors [48].

The reciprocal association between pTau and neuronal hyperexcitability appears to be particularly significant in AD with mesial temporal lobe epilepsy, posited as a distinct AD subtype [69]. Pathological studies on temporal lobe epilepsy (TLE) patients have revealed pTau-related alterations, resembling AD, which further correlate with cognitive dysfunction [56]. The stimulation of cyclin-dependent kinase 5 (CDK5) and GSK-3β has been proposed as a potential contributor to the phosphorylation of tau in TLE [48]. However, another study found that pathological findings in resected temporal lobes from TLE patients differed from those expected in AD, leading researchers to suggest that cognitive decline in TLE may be mediated by non-AD-related mechanisms [70].

Like Aβ, pTau is a potent mediator of glutamate release from presynaptic vesicles [14], suggesting a potential link between tau accumulation and cortical excitability [22]. In mouse models, pTau exerts its pro-epileptic effect by eliminating Kv4.2 K+ dendritic channels and interacting with Fyn and postsynaptic density protein 95 (PSD95). Specifically, tau’s interaction with PSD95 augments post-synaptic glutamate receptors, rendering them more susceptible to excitability. While tau phosphorylation initially aims to reduce this interaction, over time it becomes detrimental [60,67]. Furthermore, pTau is implicated in neuroplasticity, neuron migration, and neuronal network rearrangement, all of which are processes associated with epilepsy [48]. Knockout of tau in a *hAPP* mouse model resulted in a decrease in epileptiform activity in the hippocampus and in cognitive decline, while preserving normal operation of NMDA receptors and impairing long-term potentiation [64].

Vice versa, seizures trigger inflammatory processes and neuronal excitotoxicity. Tau phosphorylation moderates the extent of these harmful procedures and exerts a neuroprotective action. Evidence suggests that total tau levels gradually return to normal approximately four months after status epilepticus in animal models [4,58]. However, this prolonged situation may eventually reach a threshold where tau phosphorylation becomes toxic. In an attempt to mitigate this effect, Aβ accumulates to mediate cell death through excitotoxicity, but this mechanism fails, potentially due to the involvement of the endoplasmic reticulum. As a result, more pTau and Aβ aggregate, leading to pathologic elements reminiscent of AD [58]. This hypothesis highlights the importance of prompt therapeutic intervention. Indeed, elevated levels of pTau are associated with neuronal damage and hippocampal atrophy [48].

Notably, total tau levels, but not pTau and Aβ42 levels, were associated with seizure onset in AD patients, suggesting a cortical effect of tau protein. Decreased total tau levels were associated with reduced hyperexcitability and seizures and prevented impairment in memory and learning in mouse models [48,60]. A proteome study uncovered a trend toward altered expression of common proteins involved in axonal, synaptic, microtubular, and mitochondrial function in both epilepsy and AD. Many of these alterations may be influenced by Tau oligomers, placing Tau at the epicenter of the association between these two conditions [59].

#### 3.3.3. Glutamate

Glutamate, the primary excitatory neurotransmitter in the central nervous system (CNS), plays a pivotal role in the pathogenesis of both AD and epilepsy. Dysregulation of glutamate signaling is a key pathway implicated in both conditions [58]. Alongside the effects of Aβ and tau pathology on glutamate levels, mutations in glutamate receptors such as NMDA and AMPA have been observed in AD, contributing to an increased predisposition to epilepsy [57]. Moreover, research has shown that severely affected AD patients exhibit decreased levels of glutamate compared to those with milder forms of the disease. This imbalance in glutamate levels contributes to the dysregulation of excitatory and inhibitory activity in the brain [58]. The dysfunction of the NMDA glutamate receptor in AD can contribute to excitotoxicity by promoting the formation of amyloid plaques, hyperphosphorylated tau, and by inducing neuronal death [14]. Simultaneous depolarization of glutamatergic neurons increases the risk of epileptic activity [61]. Meanwhile, epilepsy is associated with an increased quantity of NMDA and AMPA receptors. When overstimulated, these receptors trigger the formation of free radicals, leading to oxidative stress and mitochondrial dysfunction, which can insult neuronal cells in the long term, potentially resulting in cognitive decline [4].

#### 3.3.4. Gamma-Aminobutyric Acid

GABA signaling serves as the primary inhibitory component of the CNS. Anomalies in GABA signaling have been shown to precede disruptions in glutamate signaling, tipping the balance toward excitatory inputs [57]. Studies have found lower levels of GABA_A_ receptors in patients with severe AD compared to those with mild AD, and cortical neurons neighboring Aβ plaques exhibit loss of many GABAergic terminals [4,58]. Impairment of GABAergic interneurons, particularly in the hippocampus, has been observed in AD [14] along with suggested loss of GABAergic synapses [50]. In both the CSF and temporal cortex of AD patients, total GABA levels are significantly reduced, accompanied by decreases in levels of glutamic acid decarboxylase (GAD) 65/67, GABA_A_ receptors, and GABA transporters [57,71]. Activation of D1 receptors on GABAergic inhibitory interneurons results in reduced GABA release [50], while voltage-gated sodium channels may also be affected by GABA dysregulation [4]. These alterations collectively diminish the inhibitory effect of GABA, contributing to neuronal hyperexcitability and epilepsy as well as memory and learning deficits [57].

#### 3.3.5. Neuroinflammation

Neuroinflammation is strongly implicated in the pathogenesis of both AD and epilepsy [7,62,72]. Aβ directly affects astrocytes and microglia, activating these glial cells [73], while Aβ and tau protein tangles induce oxidative stress, further attracting microglia [73]. Continuous stimulation of glial cells results in the production of proinflammatory cytokines, which promote seizures by inducing glutamate release. Subsequently, seizures can exacerbate neuroinflammation, creating a cycle of pathological interaction. Elevated levels of tumor necrosis factor-α (TNF-α) have been observed in the brains of patients with both AD and epilepsy. This cytokine exerts an excitatory effect by increasing the sensitivity of AMPA and NMDA receptors while reducing GABA_A_ receptor function. Similarly, interleukin (IL) 1β impacts seizure susceptibility by reducing GABA concentration in the cortex and hippocampus, promoting glutamate release, and increasing tau phosphorylation [4,73,74]. Additionally, IL-6 has been shown to decrease seizure threshold.

Upregulation of Toll-like receptor 4 (TLR4) in the hippocampus has been observed, with its activation leading to increased influx of Ca^2+^ through the NMDA receptor, thereby elevating the risk of epilepsy. Neuroinflammation also contributes to oxidative stress and the release of more pro-inflammatory mediators, which can impair astrocyte function and promote glutamate release [4,73]. Furthermore, Aβ induces the activation of the leucine-rich repeat pyrin domain containing 3 (NLRP3) inflammasome, which is present in plaque-associated microglia and sustains a chronic inflammatory state [74]. These pro-inflammatory molecules not only contribute to neuroinflammation but also stimulate further production and accumulation of Aβ. Additionally, microglia can induce T-cell neurotoxicity, exacerbating neurodegeneration [73].

Conversely, mitigating tau accumulation and neuroinflammation has been shown to reduce seizure severity, improve cognition, and decrease mortality in mouse models of epilepsy [73]. Sustained neuroinflammation disrupts BBB integrity, which further maintains a pro-inflammatory environment, promotes Aβ production, and induces excitatory effects [27].

#### 3.3.6. Acetylcholine—Noradrenaline—Serotonin Activity

Acetylcholine is a neurotransmitter involved in memory, learning, and executive functions [4,75]. In AD, cholinergic activity progressively degenerates, leading to acetylcholine deficiency. The severity of memory loss is associated with the extent of synaptic loss in cholinergic pathways [75]. Disruption of cholinergic function in the CNS may further drive additional pathological hallmarks of AD, including tau protein phosphorylation, which, as discussed earlier, plays a pivotal role in epileptogenesis [75]. However, in the early stages of AD, before the degeneration of cholinergic pathways occurs, increased cholinergic activity may render individuals susceptible to seizures [4,14]. This heightened activity has been shown to increase excitability and subclinical epileptiform activity in animal models [4]. Cholinergic signaling is involved in regulating neural excitability. Wang et al. extensively analyzed the significance of alterations in acetylcholine receptors, cholinergic neurons, and cholinergic circuits in various epilepsy models [76].

Despite noradrenaline’s anti-epileptic effects, the early degeneration of the locus ceruleus—the most prominent noradrenergic nucleus of the CNS—in AD leads to inadequate levels of noradrenaline. This deficiency fails to counteract hyperexcitability in the hippocampus, thereby increasing susceptibility to seizures [27]. The importance of noradrenaline’s anti-epileptic effect is further supported by the detection of increased extracellular levels of noradrenaline in the mesial temporal lobe in epilepsy patients, brain samples, and during limbic seizures in rats. This increase may serve as a compensatory mechanism. Additionally, reduced noradrenergic signaling induces neuroinflammation, which, as discussed earlier, impacts epileptogenesis [77].

Serotonin is a neurotransmitter involved in memory, cognition, and mood and is suggested to play a role in the association between AD and epilepsy [19,78]. Serotoninergic signaling was found to be downregulated by 40% in AD patients [78]. Additionally, the interplay between serotonin metabolism and mitochondria in serotonin-producing neurons may be disturbed in AD, further exacerbating pathological alterations relevant to the disease [79]. Modulation of serotonin receptors 5-HT_2_R and 5-HT_4_R may inhibit Aβ production [79]. The levels of the 5HT_2_bR receptor were elevated in the brains of AD patients and antagonism of this receptor was demonstrated to have a beneficial effect on memory and synaptic plasticity [80]. Moreover, in an AD mouse model, stimulation of the serotonin receptor 5-HT_6_R was demonstrated to improve cognitive dysfunction [78]. A thorough review on serotonin signaling pathways and the effects of modulation of serotonin receptors in AD was published by Eremin et al. [81].

Dysregulation of serotonin signaling may act as one of the most important mechanisms for epileptogenesis and blocking the 5-HT_3_R receptor was proved to have both an anti-epileptic effect and a memory-beneficial effect [82]. Serotonin is suspected to be involved in sudden unexpected death in epilepsy (SUDEP), possibly due to the capacity of serotonin neurons to respond to systemic CO_2_ increases and drive proper respiratory function. In patients with temporal lobe epilepsy, 5-HT_1_A levels were reduced in the hippocampus, temporal cortex, amygdala, and frontal lobe ipsilateral to the epileptic activity, leading to a decline in the serotonin-induced anti-epileptic effect [83]. Disruption of serotonin pathways, driven by neuronal hyperexcitability, interferes with microglia, ultimately leading to increased neuroinflammation and exacerbation of AD pathology through the increased production of Aβ [19].

#### 3.3.7. Voltage-Gated Ion Channels

The quantity and activity of voltage-gated ion channels (Na^+^, Ca^2+^, K^+^) are modified in both AD and epilepsy [14]. These alterations are largely attributed to genetic factors or endogenous molecules that disrupt the balance between excitatory and inhibitory signals, thereby contributing to epileptogenesis or promoting the aggregation of pathological proteins leading to AD [72]. According to a genetic study, the differential expression of *SCN2A*, *GRIA1*, and *KCNJ9*—genes that encode the α2 subunit of the neuronal sodium channel, the AMPA-type subunit 1 receptor, and the G protein-activated inward rectifier potassium channel 3, respectively—is consistent in both AD and epilepsy. These findings suggest that these genes may be implicated in the pathogenesis of both diseases, making them potential targets for future therapeutic interventions [72].

Additionally, reduced activity of hyperpolarization activated cyclic nucleotide gated potassium channel 1 (HCN1) in the prodromal stages of AD, observed in the hippocampal CA1 and the temporal cortex, represents one of the most consistent alterations in the AD proteome. This deficit in HCN1 function leads to hyperexcitability and may contribute to the development of epilepsy, further exacerbating Aβ production. This evidence provides insight into the therapeutic effect of lamotrigine [84].

Further alterations common to both AD and epileptic syndromes have been identified in animal models, including increased Nav1.6, decreased Nav1.1 in GABAergic inhibitory interneurons, increased L-type Ca^2+^ channels, and decreased levels of calbindin, which is implicated in intracellular calcium transport in the dentate gyrus [27].

#### 3.3.8. Mitochondria–Endoplasmic Reticulum Stress

In AD, the decreased distribution of mitochondria along the axons and dendrites promotes neuronal excitability [14]. Furthermore, continuous activation of glutamate receptors triggers oxidative stress, inducing mitochondrial dysfunction and contributing to the initiation and progression of epilepsy by provoking apoptosis [4,56]. Additionally, aberrantly folded Aβ and Tau proteins accumulate in the endoplasmic reticulum, leading to endoplasmic reticulum stress and disruption of the interaction between endoplasmic reticulum and mitochondria [85].

#### 3.3.9. Astrocytes

Astrocytes play a crucial role in glutamate reuptake, a process hindered by Aβ oligomers. Additionally, astrocytes regulate extracellular potassium levels. Dysregulation of this process can lead to elevated extracellular potassium, which coincides with seizure onset. Studies have shown a reduction in the potassium channel Kir4.1 on astrocytes in AD patients. Reactive astrogliosis, characterized by astrocyte enlargement and proliferation in response to neuronal death and synaptic remodeling, further contributes to potassium homeostasis disruption [4].

#### 3.3.10. Beta-Secretase 1

Beta-secretase 1 (BACE1) is known for its involvement in producing Aβ, a hallmark of AD. However, it also plays a role in epileptogenesis by cleaving the β2 and β4 subunits of voltage-gated Na^+^ channels. This action induces hyperexcitability, potentially contributing to the development of epilepsy [21].

#### 3.3.11. Mechanistic Target of Rapamycin

Mechanistic target of rapamycin (mTOR) is widely expressed in the CNS and plays crucial roles in axon formation, synapse formation, and neuronal excitability. Its involvement in autophagy is particularly significant. Studies in animal models and cell cultures have shown that Aβ and GSK3β (an enzyme involved in Tau phosphorylation) activate mTOR, leading to reduced autophagy. Recently, autophagy has been implicated in regulating neuronal excitability by clearing Aβ and pTau. Therefore, the role of mTOR signaling in AD and epilepsy is currently under investigation. Postmortem studies of AD cases have shown increased mTOR activation along with autophagy dysfunction. Additionally, epilepsy has been shown to activate mTOR [4,27]. Inhibiting mTOR in mouse models has been found to alleviate cognitive decline and pathological features of AD [26].

#### 3.3.12. Triggering Receptor Expressed on Myeloid Cells

TREM2 is a receptor expressed by microglia, influencing various functions of these cells. Mutations in TREM2 have been associated with an increased risk of AD. Studies using TREM2 knockout mice have shown that these animals experience more focal to bilateral tonic-clonic seizures, along with reduced microglial proliferation and impaired phagocytosis. Similarly, impaired microglial phagocytosis has been observed in human cases of drug-resistant focal epilepsy [4,86]. Overall, reduced TREM2 function hampers microglial ability to regulate hyperexcitability in the brain [35]. Consequently, researchers are currently exploring the manipulation of TREM2 function as a potential novel therapeutic approach [4,86].

#### 3.3.13. α-Synuclein

α-synuclein is involved in synaptic function and neuronal plasticity, but it also contributes to neuroinflammation. In AD, α-synuclein pathology may be evident and negatively affects cognition [87]. Research indicates its involvement in epilepsy, where its presence has been associated with neuronal loss, reactive gliosis, and increased severity of epilepsy. Consequently, α-synuclein may contribute to epileptogenesis in AD by activating glial cells, promoting neuroinflammation, and inducing oxidative stress [4].

#### 3.3.14. Neural Network

Normal neural network activity is essential for regulating various brain functions, including memory, by finely tuning electrical activity and its fluctuations. Evidence suggests that disruption of neural network activity occurs decades before the clinical onset of AD [27] and contributes to synaptic and cognitive deficits [42]. This disruption may represent a fundamental aspect of AD mechanisms and indicates that epileptic activity may be an integral part of the AD phenotype [42].

#### 3.3.15. The Role of Sleep

Sleep is recognized as an important factor in linking AD and epilepsy. Approximately 40% of AD patients experience sleep disruption [88] with insomnia and sleep apnea identified as risk factors for AD development [89]. AD itself induces sleep fragmentation and reduces REM and overall sleep duration, alterations that may contribute to the accumulation of Aβ and tau pathology. This relationship appears to be reciprocal, as sleep deprivation leads to increased CSF Aβ levels. In this case, levetiracetam was observed to be effective in an animal model. Furthermore, tau kinetics during sleep and wakefulness resemble those observed in Aβ. The glymphatic system, which operates predominantly during sleep, plays a crucial role in clearing tau and Aβ from the brain. Reduced sleep in AD may hinder the glymphatic system’s function, leading to the worsening of AD pathology [89].

During sleep, the procedures of memory consolidation take place. However, epileptic abnormalities, attributed to hippocampal ictal or interictal activities are also increasingly occurring during sleep due to the same procedures. This interferes with normal memory consolidation and leads to cognitive decline [60] and further fragmentation of sleep [89]. EEG monitoring of AD patients during sleep has revealed subclinical seizures [90].

It is evident that AD disrupts normal sleep patterns, which can facilitate the onset of epileptic activity, thereby further negatively affecting cognition and creating a vicious cycle. Hanke et al. suggest that perampanel may have a beneficial effect in regulating these interactions, while trazodone in combination with levetiracetam could also be effective. Additionally, gabapentin has shown positive effects on both sleep and epilepsy. Neuromodulation through transcranial current stimulation during sleep is currently being studied as a method aiming at improving memory [89].

#### 3.3.16. Others

Alterations at the cellular level are also reported. In AD, dendrite length is reduced, allowing neurons to generate action potentials at a lower threshold [14]. Additionally, aberrant neurogenesis has been reported in the frontal, temporal, and entorhinal cortex in AD patients with SEA. Epileptic AD patients have also shown neuronal loss in the parietal cortex and parahippocampal gyrus, as well as gliosis of the temporal lobe. These alterations may contribute to epilepsy or, alternatively, result from its occurrence [13,50]. AD causes focal epilepsy with hippocampal sclerosis, whereas less robust data support the reverse association as well. In fact, the hippocampus and mesial temporal lobe are the primary loci where network hyperexcitability and seizures emanate from [91]. Additionally, evidence suggests a reciprocal causal relationship between AD and focal epilepsy with hippocampal sclerosis, as confirmed by a mendelian randomization study [56].

The choroid plexus is a region prone to pathological alterations in both AD and epilepsy, including Aβ accumulation, inflammation, apoptosis, vascular changes, and glial cell metabolism. Further research focusing on the choroid plexus could provide valuable insights into the connection between AD and epilepsy [26].

Epileptic activity has been linked to the downregulation of calbindin, which plays a role in calcium homeostasis and prevention of neuronal excitability, as well as Fos, which is involved in synaptic plasticity and memory formation, ultimately leading to cognitive decline [92]. Additionally, status epilepticus has been shown to predispose individuals to AD by causing hippocampal neuronal cell loss, a process mediated by nuclear factor kappa-light-chain-enhancer of activated B cells (NF-κΒ) produced by macrophages [93]. As reported, upregulation of GSK3β, a factor associated with AD, contributes to seizures by increasing phosphorylation of Tau or activating other Tau kinases. Furthermore, GSK3β may stimulate Fyn-mediated NMDA receptor activation and Ca^2+^ influx [94]. Transcriptome analysis has revealed an increase in Frizzled Class Receptor 7 (FZD7) levels in the temporal cortex, providing further insight into potential therapeutic implications [95].

Another speculative mechanism involves the disinhibition of thalamic relay nuclei, which are responsible for the projection of signals to the cortex due to anomalies of the cortical input to the reticular thalamic nucleus [14]. Additionally, allopregnanolone, an endogenous neurosteroid, is observed to be diminished in individuals with AD, potentially resulting in inadequate neuroprotection. Moreover, sustained depletion of allopregnanolone levels may trigger glial cell activation, promoting the generation of neurotoxic substances and facilitating seizure activity [21]. Finally, in an AD mouse model, early seizures were observed to stimulate the proliferation of neural stem cells, whereas recurrent seizures depleted them, ultimately resulting in cognitive impairment [96].

### 3.4. Seizure Types

LOE typically presents with focal impaired awareness seizures without overt motor symptoms in 66–78% of cases, with status epilepticus occurring in approximately 11% [25,27]. Other described seizure types include focal to bilateral tonic-clonic seizures, focal seizures without impaired awareness, and generalized seizures (less than 15% of cases) [7].

Previous studies initially suggested that up to 89% of seizures in AD patients were generalized tonic-clonic seizures, primarily thought to arise from focal to secondary generalized seizures [11]. However, with the implementation of extended EEG recordings, it has become evident that focal seizures with impaired awareness are the predominant type [56,91]. More than half of these seizures are non-convulsive [56] and may present with subtle symptoms such as déjà vu, jamais vu, unprovoked emotions, altered responsiveness, confusion, automatisms, and sensory episodes (such as a metallic taste or epigastric ascending sensation), or with staring, speech interruption, and memory impairment [11,15,66]. Transient epileptic amnesia is a rare type [11]. Subclinical seizures pose a significant concern as they are challenging to detect and are strongly associated with cognitive decline [97].

In early-onset AD patients, seizures were primarily characterized as tonic-clonic, typical temporal seizures, and myoclonus, with additional occurrences of focal onset extra-temporal and other types [20]. However, in advanced AD, approximately 15–40% of patients experienced generalized tonic-clonic seizures, while focal seizures accounted for approximately 70% of the seizures they encountered [9].

### 3.5. Electroencephalography

EEG findings have garnered significant interest in understanding the pathogenesis and progression of disease [63]. They hold potential to extend beyond the amyloid and tau hypotheses, which have so far fallen short in producing adequate AD treatments [98]. Contrary to traditional assumptions attributing cognitive impairment in AD to reduced synaptic activity, recent evidence from AD animal models, in vitro experiments, and human studies suggests that neuronal hyperactivity results from disruption in excitatory-inhibitory balance. This imbalance, once induced by Aβ in conjunction with other processes, particularly in the early stages of AD, triggers synaptic failure, memory dysfunction, and neurodegeneration in a self-perpetuating manner [61]. This neuronal hyperexcitability of the cerebral cortex can manifest as seizures or subclinical epileptiform discharges [99]. Characteristic EEG findings indicative of neuronal hyperexcitability in patients with AD include epileptiform discharges, small sharp spikes, temporal intermittent rhythmic delta activity (TIRDA), and paroxysmal slow cortical activity [99].

In a recent study by Lam et al., utilizing 24-h ambulatory EEG monitoring, it was observed that 22% of participants with AD without epilepsy and 53.3% of those with AD and epilepsy exhibited epileptiform EEG activity. There was a notable contrast in the severity of these abnormalities between the two groups. In the AD epilepsy group, epileptiform discharges were evident in both the left and right temporal regions, indicating widespread network disturbances. Additionally, epileptiform discharges were most frequently observed during N2 sleep across all groups [100].

Small sharp spikes, also known as benign sporadic sleep spikes, typically manifest during early drowsiness and stages N1 and N2 of sleep [99]. Recent research has highlighted their significance, particularly in the context of AD. Unilateral small sharp waves with high frequency have been linked to clinical seizures in AD [100].

TIRDA is observed in approximately 26% of AD patients. While TIRDA is more likely to occur during N2 sleep and in the left temporal region, its occurrence during awake or REM states is strongly associated with seizures in AD [100].

In EEG recordings of AD patients, diffuse slowing has been observed in approximately 38% of cases, while focal slowing is present in around 20% [50]. Milikovski et al. have identified a component of cortical slowing termed paroxysmal slow wave events, characterized by transient paroxysmal slowing of the cortical network, with a median power frequency lower than 6 Hz sustained for at least 5 consecutive seconds on scalp EEG recordings. Detection of these events in routine scalp EEG may serve as a diagnostic indicator for subclinical seizures [46,99].

Importantly, clinically observed epilepsy represents just one aspect of the larger picture. Evidence suggests that epileptic activity related to AD is primarily subclinical, with over 40% of AD patients displaying such subclinical epileptic activities, which may be apparent early in the disease course, along with hippocampal network hyperactivity [11,28,99]. These activities are defined as paroxysmal sharp waveforms lasting 20 to 200 ms and disrupting background activity. Detecting SEA and interictal epileptiform discharges (IEDs) can be challenging with conventional methods, prompting the use of overnight long-term monitoring with video-EEG (LTM-VEEG) or magnetoencephalography with simultaneous EEG (M/EEG) to improve detection [11,60]. In a recent study, SEA was found in 50% of AD dementia patients, 27% of MCI due to AD patients, and 25% of preclinical AD subjects [13].

Effectively recognizing epilepsy in AD patients poses a challenge due to potential subclinical or non-convulsive epileptic activity overlapping with AD symptoms. Routine EEG is infrequently used in AD patients [4,60,62], and even when used, epileptic activity may not be readily apparent due to localization within the hippocampus and mesial temporal lobe, regions primarily affected by AD and common sources of focal epilepsy in this population [26,97]. Consequently, epileptic activity may go unnoticed for a significant period [60]. Recording time by scalp EEG is highly significant for the detection rate of epileptic discharges. Only 3% of patients with AD display epileptic discharges in 20 min of eyes-closed EEG recordings. Serial EEGs, 8-h sleep EEG, and ambulatory EEG studies seem to provide greater diagnostic value for detecting epileptiform activity [99]. Long-term ear-EEG monitoring has also proven useful in detecting epileptiform discharges, especially spikes originating from temporal lobes occurring at night [101]. High-density (Hd) EEG has been used in a study to evaluate the potential added value of the inferior temporal chain in AD patients, concluding that combined with long-term monitoring EEG, these examinations could lead to a higher detection of SEA [13]. In another study, Hd EEG detected EEG spikes in 46% of aMCI patients, revealing early signs of hyperexcitability [22]. Given that, clinical evaluation of AD patients is crucial for successfully implementing the appropriate diagnostic workup, and continuous follow-up is essential to ensure that epileptic activity is effectively detected and treated.

### 3.6. Biomarkers

**Figure 3:**
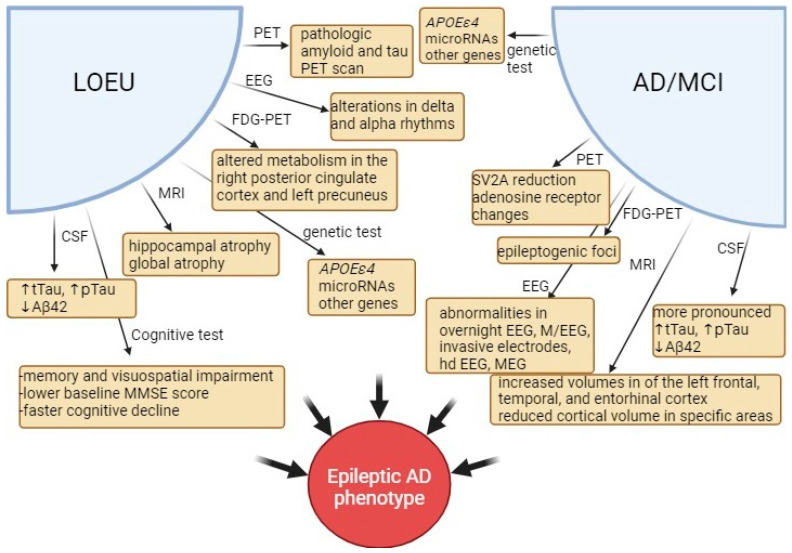
Suggested biomarkers for the comorbidity of Alzheimer’s disease and late onset epilepsy of unknown etiology. LOEU: late-onset epilepsy of unknown etiology; AD: Alzheimer’s disease; MCI: mild cognitive impairment; PET: positron emission tomography; EEG: electroencephalogram; FDG-PET: fluorodeoxyglucose positron emission tomography; MRI: magnetic resonance imaging; CSF: cerebrospinal fluid; APOEε4: apolipoprotein ε4 allele; tau: Tau protein; tTau: total Tau protein; pTau: hyperphosphorylated Tau protein; Aβ42: amyloid-beta 42; MMSE: Mini-Mental State Examination; SV2A: synaptic vesicle glycoprotein 2A; M/EEG: magnetoencephalography with simultaneous EEG; hd EEG: high density EEG; MEG: magnetoencephalography.

It is obvious that the pathologic process linking AD and epilepsy begins before clinical symptoms manifest. Therefore, the implementation of biomarkers becomes crucial. Since established damage to the CNS is challenging to treat, these biomarkers should identify at-risk patients early enough for intervention to potentially delay, halt, or even reverse disease progression. Novel treatments for AD currently under research focus on targeting this early stage, and research into anti-epileptic treatments also emphasizes early initiation of anti-seizure medications. Thus, precise and early identification of eligible patients is crucial for maximizing treatment benefits. Currently, multiple approaches are used to explore potential biomarkers, and some of the suggested biomarkers are depicted in Figure 3.

#### 3.6.1. Cerebrospinal Fluid Biomarkers

Recent studies have highlighted a connection between seizures in AD patients and more pronounced deviations in CSF AD biomarkers [59]. Specifically, epileptic AD patients exhibited elevated levels of tTau and pTau along with reduced levels of Aβ42 compared to AD patients without epilepsy [68]. Additionally, low CSF Aβ42 levels associated with genetic factors relevant to AD contributed to an increased risk of generalized epilepsy [91]. Another study involving AD patients revealed that those with epilepsy displayed increased CSF Aβ40 levels associated with delta wave slowing and increased CSF Aβ42 levels associated with IEDs. While these measures were not definitive for epilepsy diagnosis, elevated CSF Aβ40 levels could suggest that AD patients are at an increased risk for epilepsy. The distinct effects of Aβ40 and Aβ42 were proposed to be responsible for their association with different EEG patterns [41].

In patients with LOEU, reduced CSF Aβ42 levels and increased tTau or pTau levels compared to controls are associated with an increased risk of dementia within the following three years [7,65]. The risk of LOEU increases following a decrease in the ratio of serum Aβ42 to Aβ40 during the transition to late adulthood [102]. This suggests that the rate of amyloid aggregation, rather than total amyloid quantity, may be implicated in LOEU risk [8]. Furthermore, CSF neurofilament light chain (Nfl) and glial fibrillary acidic protein (GFAP) values were not significantly different in AD patients with or without epilepsy, suggesting that epilepsy is specifically associated with AD pathology rather than other neurodegenerative alterations affecting these markers [68]. LOEU patients with MCI have decreased CSF Aβ42 compared to LOEU patients who are cognitively intact [103]. Additionally, elevation of CSF proteins, including neurogranin, synaptosomal associated protein 25 (SNAP25), synaptotagmin, growth-associated protein 43 (GAP-43), and TREM2, may aid in the identification of AD-related mesial temporal lobe epilepsy [104].

It is evident that these markers are promising in the field of AD and epilepsy, as they can aid in prognosis. Measuring AD-related parameters in the CSF of patients with LOEU may help identify a subgroup of patients at risk for AD before clinically apparent cognitive decline. Conversely, another subgroup of AD patients predisposed to seizures may be identified if a specific range of marker values is implemented. In that case, prompt initiation of treatment could achieve beneficial effects.

#### 3.6.2. Neuroimaging Findings

Neuroimaging findings on magnetic resonance imaging (MRI) useful for identifying early-stage AD include hippocampal atrophy [47]. In AD patients with SEA, volumes of the left frontal, temporal, and entorhinal cortex were found to be larger than those in AD patients without SEA. These structural differences were particularly pronounced in brain regions where IEDs had been recorded [13]. In epileptic AD patients, MRI reveals reduced cortical volume in specific areas such as the right inferior parietal lobule, left lingual gyrus, and cerebellar region when compared to non-epileptic AD patients. Conversely, these patients exhibit larger volumes in regions like the left middle frontal, postcentral, and right middle temporal areas. The cortical areas with decreased volumes in epileptic AD patients are predictive of cognitive decline. Researchers suggest that both AD and seizures impact cortical volumes and cognition [105]. A study involving patients with TLE found that median temporal lobe atrophy on MRI volumetric analysis was similar to that of patients with aMCI and both groups exhibited common cognitive deficits, shedding light on the increased risk of epileptic patients for AD [15]. From a technical perspective, it would be interesting to explore the temporal coincidence of these changes’ onset with the clinical appearance of epilepsy to decipher their prognostic role. TLE and aMCI overlap in the characteristics of brain atrophy [106]. Despite these differences, MRI findings alone may not be sensitive enough to reliably distinguish epileptic AD from non-epileptic AD patients [107]. Functional MRI (fMRI) utilized in the early stages of AD helps identify dysfunction across broad brain areas, and detect short-duration abnormalities [108]. In LOEU, MRI findings commonly encompass global atrophy, temporal, uni- or bi-lateral hippocampal atrophy, and frequently observed white matter hyperintensities [7]. Volumetric MRI studies in epilepsy often reveal patterns of temporal and parietal atrophy [41]. These patterns resemble those observed in AD and could reflect common underlying mechanisms. Future studies are needed to better standardize these findings and identify their role in the routine screening of AD or LOEU patients based on the potential clinical benefit.

Findings from fluorodeoxyglucose (FDG)-positron emission tomography (PET) are superior to other diagnostic methods in diagnosing AD. In early-stage AD, patients exhibit hypometabolism in the temporoparietal area, posterior cingulate cortex, and precuneus [47,109]. FDG-PET scans conducted in patients with LOEU reveal altered metabolism, particularly in the right posterior cingulate cortex and left precuneus, which correlates with poorer recall performance. The overlap of these metabolic alterations between the two conditions likely reflects common disease processes and should be considered in clinical evaluations. Additionally, reduced metabolism in the temporal lobe, often lateralized, concurs with abnormal lateralized EEG findings [7]. FDG-PET is also useful for detecting epileptogenic foci [108]. Advanced PET techniques are expected to aid in reliably identifying the comorbidity of AD and epilepsy. PET imaging methods such as [^11^C]UCB-J PET can visualize reductions in synaptic vesicle glycoprotein 2A (SV2A) in the hippocampus, suggesting synaptic dysfunction in AD [110]. PET can also detect changes in adenosine receptor expression in AD. Pathologic amyloid and tau PET scans can provide timely information valuable for identifying the underlying pathology in LOEU leading to AD [7].

#### 3.6.3. Cognitive Testing

Neuropsychological evaluation is valuable in epileptic AD patients, revealing deterioration not only in memory but also in visuospatial function and daily activities, with a more pronounced decline in language and visuospatial function observed one year later [41]. Visuospatial impairment strongly correlates with epileptic activity at AD onset, with parietal lobe atrophy, particularly in the precuneus, implicated in this association [111]. Baseline MMSE scores are lower in AD patients who later experience epileptic seizures [52]. Epileptic MCI patients exhibit multiple cognitive abnormalities compared to non-epileptic MCI patients [7,103]. Among individuals with MMSE scores >24, LOEU patients show worse performance in recall, verbal fluency, and executive function, with worsening memory deficit, decreased MMSE scores, and poorer recall observed after 12 months [7]. The Montreal cognitive assessment test is identified as an effective screening tool for cognitive decline in epilepsy [112]. Thorough cognitive testing should not be omitted in LOEU and in the early stages of AD to establish a baseline and identify specific areas of cognitive impairment. Cognitive follow-up should be implemented for patients showing signs of cognitive decline following a comprehensive diagnostic evaluation, ensuring close monitoring.

#### 3.6.4. Genetic Associations

Genetic markers serve as early biomarkers and can provide meaningful information. In certain diseases, such as breast cancer, presymptomatic genetic testing can significantly influence diagnostic and treatment strategies. As reported earlier, multiple genes are possibly implicated in AD and LOEU, as well as in their association, and could be used as early biomarkers. Asymptomatic young adult carriers of mutations relevant to autosomal dominant AD or the *APOEε4* allele, as well as asymptomatic adults with an increased risk of late-onset AD due to genetic factors, exhibit apparent task-related hippocampal hyperactivity, suggesting shared mechanisms. This hyperactivity could potentially serve as a marker for early detection of AD risk in these individuals [91]. Additionally, the identification of subclinical epileptiform activities in *APOEε4* carriers may further aid in detecting individuals at risk for early-onset AD [113]. MicroRNAs also show promise as biomarkers for both AD and epilepsy, as alterations in these molecules are commonly observed in these and other neurodegenerative diseases [108,114]. MicroRNAs are important for central nervous system development and function. Common, aberrantly expressed microRNAs between AD and epilepsy include miR-21-5p, miR-29c-3p, and miR-124-3p, which are downregulated in the CNS and miR-146a-5p and miR-223-3p, which are upregulated in the CNS. Many of these microRNAs may interfere with inflammation, Aβ production, autophagy, apoptosis, and microglia activation [115]. Genetic studies have provided valuable insights, revealing differentially expressed genes in epilepsy and AD. For instance, impairments in multiple endocytosis-related pathways and downregulation of regulator genes involved in circadian rhythms have been observed [116]. Furthermore, genes such as *SCN3B*, *EPHA4*, GABRB3, and *SCN2A* may play roles in the development of epilepsy in the context of AD [117]. These genetic findings contribute to our understanding of the molecular mechanisms underlying the association between epilepsy and AD and could be used as potential biomarkers. In order to achieve this, large studies should be undertaken in order to explore the extent to which each of them increases disease risk and the implications for future medical management of the individuals.

#### 3.6.5. Electroencephalographic Markers

Epileptic activity in AD is an under-recognized entity given its subclinical characteristics and the fact that about 85% of standard EEG recordings are reported to be normal [41]. This underscores the need for more sensitive techniques to detect abnormalities in brain activity. Advanced methods such as overnight EEG, M/EEG, and invasive electrode placement through the foramen ovale offer increased sensitivity compared to standard EEG [41]. For instance, overnight Hd EEG with 256 channels revealed hyperexcitability in 46% of aMCI patients, a finding not detectable by other methods such as imaging or neuropsychological assessment at such an early stage of the disease [22]. Combining standard EEG with longer duration recordings and magnetoencephalography (MEG) has proven effective in identifying abnormalities in 42.4% of AD patients who had never experienced seizures [22,118]. Interestingly, even mild dementia patients without seizures showed improved memory and executive function with low doses of levetiracetam, suggesting a potential therapeutic benefit in this population. These findings underscore the importance of better characterizing MCI/AD patients who may benefit from such interventions [22]. MEG demonstrates sensitivity in detecting abnormalities early in the course of temporal lobe epilepsy and AD, particularly within the theta and gamma rhythm ranges [108]. Foramen ovale electrodes serve as a valuable method for observing hippocampal epileptic activity even in the absence of clinical signs early in AD progression [108].

In animal models, a potential EEG biomarker for the progression from epilepsy to AD was investigated. This marker involves high-frequency oscillations (250–500 Hz), which were identified in AD mouse models but not in control subjects [15]. Quantitative EEG shows promise in diagnosing AD, with data indicating that elevated relative theta power may be an early alteration preceding dementia and could serve as a biomarker [85,119]. Normal theta and gamma oscillations are essential for cognitive functions, particularly memory, and disturbances in these rhythms, as well as their consistency between hippocampal and cortical areas, are observed in both AD and temporal lobe epilepsy. In the future, magnetoencephalography combined with coherent analysis is expected to detect these changes and provide valuable diagnostic and treatment insights. [119,120]. On EEG, alterations in delta and alpha rhythms are detected in epileptic patients with MCI compared to non-epileptic MCI patients [103]. Additionally, a link between reduced REM sleep duration and the presence of seizure-related epileptiform activity in AD patients has been suggested [32].

### 3.7. Therapy

Anti-epileptic treatment plays a crucial role in managing AD, primarily by controlling seizures. Studies indicate that up to 90% of patients receiving treatment achieve sufficient seizure control with a single medication, regardless of the stage of AD [7,15,25,27,42,121]. Secondly, studies involving AD and MCI patients, as well as animal models, demonstrate improved cognitive outcomes with anti-epileptic treatment. Thirdly, experimental findings in rodent models suggest that such treatment mitigates disease pathology, including hippocampal remodeling, neuronal and synaptic impairment, abnormal microglial gene expression, and even Aβ production, accumulation, and plaque formation. In cases of epileptic prodromal AD, treatment with anti-epileptic drugs has led to cognitive outcomes similar to those of non-epileptic AD patients [28,35,122]. This data supports the notion that regulating neuronal hyperexcitability not only improves cognition but also underscores the association between epilepsy and AD.

Levetiracetam (LEV) has recently been attributed all these beneficial effects and has emerged as a promising and effective treatment [28]. LEV binds to synaptic vesicle glycoprotein 2A (SV2A) and, in animal models, regulates glutamate release from astrocytes, mitigates synaptic impairment (leading to suppression of IEDs), and improves cognitive symptoms [63,123]. Moreover, according to mouse experiments, LEV may inhibit tau phosphorylation, reduce neuroinflammation by deactivating inflammasomes, restore proper mitochondrial function, and aid in Aβ degradation by enhancing autophagy. However, it is worth noting that high levels of LEV were found to be toxic to neurons [27,74]. In AD, LEV has been shown to ameliorate activity in the CA3 hippocampal area and dentate gyrus, improve cognitive dysfunction (such as spatial memory and executive function) in patients with epilepsy or SEA, and restore oscillatory activity. Notably, a meta-analysis found no cognitive adverse effects associated with LEV use [15,27,123,124]. In particular, non-epileptic patients with aMCI showed cognitive improvement following the mitigation of hippocampal hyperexcitability. Meanwhile, mild AD patients exhibited a dose-dependent response to treatment, with low doses leading to increased blood flow to the temporal lobe and hippocampus, and high doses normalizing brain activity. Additionally, both epileptic and non-epileptic AD patients experienced improvements in visuospatial abilities, while epileptic AD patients maintained preserved attention and language fluency. Furthermore, favorable effects on hippocampal activity, visual memory, and attention were observed in healthy elderly individuals [125]. LEV has fewer adverse events compared to other drugs, including lamotrigine, phenobarbital, and phenytoin [11]. However, behavioral changes may be a reason for discontinuation of LEV treatment [15]. In such cases, brivaracetam (BRV) could serve as an alternative, as it has been proven effective with no prominent cognitive adverse effects [126]. BRV functions by mitigating glutamate-induced excitotoxicity via its association with SV2A, and animal models have shown its ability to reverse memory impairment [9,27].

Lamotrigine (LAM) is another commonly prescribed medication that has demonstrated efficacy similar to LEV. It is shown to be well-tolerated and positive effects on mood have been reported [15,62]. However, myoclonus may be exacerbated [42]. As discussed earlier, LAM acts by interacting with HCN1 channels, thereby reducing neuronal excitability [84]. Animal studies have also shown additional mechanisms of action, including the reduction of glutamate release, downregulation of BACE1 expression and mTOR signaling, degradation of Aβ plaques, and promotion of neurogenesis. It also increases the expression of B cell lymphoma-2 family of proteins (Bcl-2) to prevent apoptosis in the CA1 hippocampal region [27]. However, data on LAM’s effects on cognition are conflicting, with some studies reporting worsening cognition and a meta-analysis suggesting only a limited effect [9]. According to Rizzello et al., LEV and LAM are the most effective options for seizure control and cognitive outcomes in epileptic AD [84] making them recommended first-line treatments for various seizure types, including focal, generalized, myoclonic, and unclassified seizures [11]. LAM and topiramate (TOP) have demonstrated efficacy in suppressing IEDs [63]. Phenobarbital, TOP, and LEV were found to be equally effective in AD, nevertheless LEV was associated with better cognitive outcome and fewer adverse effects [127]. TOP and LEV may exert their effects through various mechanisms, including inhibition of histone deacetylase and anti-inflammatory and neuroprotective actions [74]. TOP has also shown efficacy in reducing Aβ deposition in AD mouse models [11]. However, TOP carries a risk for cognitive adverse effects and is generally not recommended for use in the elderly [50].

Lacosamide (LAC) has been reported as well-tolerated [15] and effective in epileptic AD patients [126], showing no negative impact on cognition [9]. LAC exerts its effects by inhibiting tau phosphorylation and histone deacetylase [27]. Perampanel has been effective in improving cognition and psychiatric symptoms in an AD patient with myoclonic epilepsy [97], and is not associated with cognitive decline [9]. Eslicarbazepine, a third-generation anti-epileptic drug, has minimal effects on cognition [66]. According to Rohracher et al., LAC and BRV are recommended as the treatment of choice in elderly patients due to their limited potential for drug interactions [128].

The impact of anti-epileptic treatments on cognition has sparked considerable debate, with numerous studies yielding conflicting results and often failing to account for important confounding factors. While overall use of anti-epileptic drugs has been linked to an elevated risk of dementia, further analysis has revealed that only certain drugs, such as valproate, carbamazepine, and clonazepam, are associated with a significant increase in dementia risk [129]. Dusanter et al. concluded that combining treatments or using valproate and topiramate together has been shown to be particularly detrimental and should be avoided [130]. In a retrospective study, treatment with sodium valproate and lamotrigine was associated with an increased risk of dementia and AD. Sodium valproate treatment was associated with a 40% increased risk for both dementia and AD after 23 years, while lamotrigine treatment specifically increased the risk of AD [131]. However, randomized controlled trials have not consistently demonstrated significant cognitive adverse effects associated with lamotrigine use [62]. Carbamazepine, oxcarbazepine, and zonisamide have been observed to affect cognition in an unclear manner, and their use should be avoided in individuals with AD. Similarly, valproate, benzodiazepines, phenobarbital, and topiramate carry a high risk of cognitive decline and are not recommended for prescription in AD patients [50,62]. Phenytoin, primidone, barbexaclone, ethosuximide, and zonisamide have also been linked to an increased risk of dementia and AD, while lorazepam has shown negative effects on various parameters including EEG, evoked potentials, and cognitive tests [15]. Despite their potential adverse effects on cognition, anti-epileptic drugs may improve cognitive function not only by reducing seizures, which are known causes of cognitive dysfunction, but also through indirect mechanisms such as stimulating neurogenesis [129]. Overall, new-generation anti-epileptic drugs are not associated with major cognitive adverse effects and are preferable for use in the elderly population [62].

AD patients often require additional treatment to manage the psychiatric complications of the disease, which may include antidepressants, benzodiazepines, and neuroleptics. However, caution is warranted as some of these medications have been associated with an increased risk of seizures. For example, trazodone, lofepramine, and venlafaxine among antidepressants, and olanzapine, quetiapine, and first-generation antipsychotics among neuroleptics have been linked to a higher risk of seizures. Clinicians should be mindful of this potential interaction when prescribing medications to AD patients [15,62].

There is a suggestion that memantine may exert beneficial effects on epilepsy in AD patients, possibly by alleviating neuroinflammation. However, clinical data supporting this hypothesis are lacking, and further trials are needed to confirm its efficacy [132]. Other glutamate antagonists targeting GluN2B and mGluR5 receptors may also hold promise in restoring the balance between excitatory and inhibitory neurotransmission [133]. Neuroinflammation is a focus of ongoing research, with nonsteroidal anti-inflammatory drugs being considered as potential interventions due to their interaction with inflammatory pathways implicated in AD and epilepsy. Studies investigating agents targeting specific inflammatory mediators such as TNF-α, TREM2, and cluster of differentiation 33 (CD33) have shown promise in AD, while inhibitors of Toll-like receptor 3 (TLR3), TLR4, and anti-high mobility group box-1 (HMGB1) have demonstrated positive effects on seizures. Future research and clinical trials will clarify the clinical utility of these novel agents [73]. Additionally, a combination of baclofen and acamprosate, both acting on GABA receptors, is being evaluated in AD patients [133]. Muscimol, a GABA_A_ receptor agonist, has proved beneficial in spatial learning and memory, while diazepam, a GABA_A_ receptor agonist, and gammapyrone could act protectively in the initial stages of AD [85]. Rapamycin, an mTOR inhibitor, had some beneficial effects in mouse models and epilepsy syndromes and may hold promise for therapeutic use in AD [134].

In the pursuit of effectively treating both AD and epilepsy, new therapeutic approaches have emerged. One strategy involves leveraging the ability of nerve growth factor to preserve the function of cholinergic neurons and neuronal networks in key regions such as the hippocampus and cortex [113]. Additionally, novel therapeutic avenues focus on inhibiting specific pathways implicated in the pathogenesis of epileptic AD. These include targeting molecules such as c-Jun N-terminal kinase (JNK) [135], and GSK3β [64], as well as inhibiting tau acetylation [64]. Tau immunotherapy is also under consideration as a potential future treatment modality. Furthermore, the utility of techniques such as deep brain stimulation and neurosurgical procedures is currently being explored as potential interventions [133].

The debate surrounding the treatment of SEA in AD patients remains contentious, highlighting the need for further clinical trials to elucidate this issue [121]. While the presence of epileptiform activity during interictal EEG does not necessarily indicate future epilepsy development, studies suggest that mitigating such abnormalities may lead to symptom improvement in AD patients [22,50]. Clinicians must consider various factors such as spike morphology, frequency, periodicity, duration, and amplitude when deciding whether to initiate treatment for SEA [63]. Ultimately, treatment decisions should be based on individual patient evaluations and require a case-by-case analysis by clinicians.

## 4. Discussion

**Figure 4:**
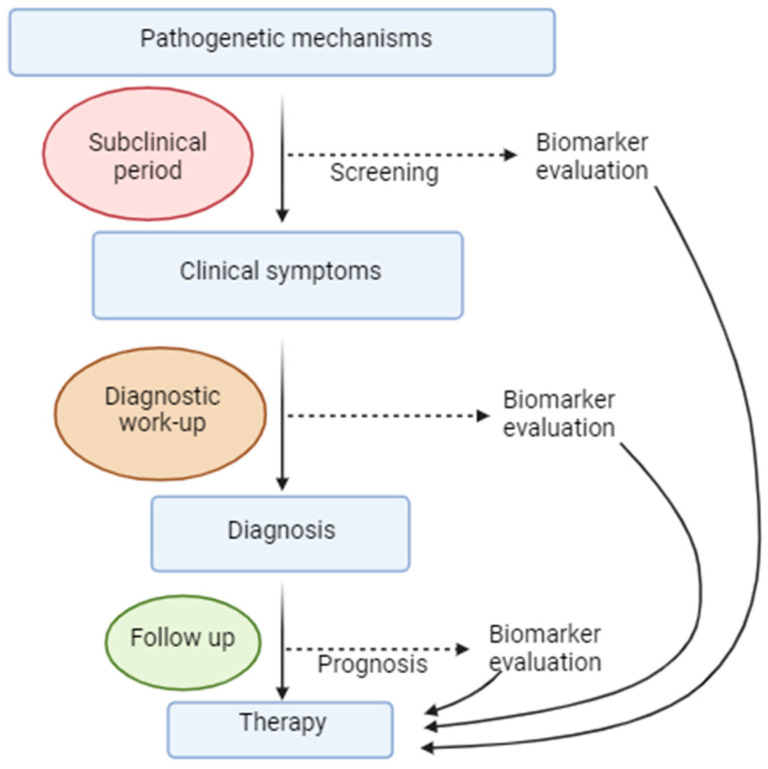
The role of biomarkers in early therapeutic intervention.

The evolving understanding of shared pathogenetic mechanisms between AD and epilepsy underscores the importance of implementing precision medicine strategies, facilitated by novel biomarkers (Figure 4). The overarching aim is to identify disease-modifying treatments for AD that can target the underlying mechanisms contributing to both cognitive decline and epilepsy. With a clearer understanding of the biological processes involved in LOEU and its association with cognitive decline, researchers can now explore targeted therapeutic approaches aimed at halting or reversing disease progression. Future clinical trials should consider epilepsy as a component of AD pathology when evaluating the therapeutic potential of new agents [42]. However, intervention must occur early in the disease process, before irreversible changes in the brain occur, as the central nervous system has limited regenerative capacity. This approach emphasizes the importance of early detection and intervention to effectively manage both AD and epilepsy.

Certain observations suggest the existence of an epileptic subtype of AD, characterized by clinical or subclinical epilepsy, IEDs, and a worse prognosis [63]. MCI is detected in 59% of individuals at the time of LOEU diagnosis, a significant finding given that 15% of individuals over 65 years with MCI will eventually be diagnosed with dementia [57]. Hickman et al. propose the terminology “epileptic preclinical AD” for LOEU patients without cognitive decline and “epileptic prodromal AD” for those with MCI, provided that all analyzed biomarkers demonstrate the presence of amyloid and tau pathology. This classification underscores the overlap between neuroimaging findings in LOEU and those observed in AD, in conjunction with other neurodegeneration biomarkers including Aβ and tau [7].

The utilization of biomarkers in the context of LOEU and its association with an epileptic prodromal AD phenotype is crucial for diagnostic purposes. However, before these biomarkers can be widely applied, standardization is necessary. For example, guidelines for identifying SEA should be established, and studies need to be designed in a structured manner to ensure consistent results. Yet, relying on biomarkers only becomes feasible once symptoms, even subtle ones, appear, indicating that pathology is already advanced (atrophy has occurred, network alterations have been installed, neuron loss has taken place) and treatment may have limited effects. Thus, there is a critical need for biomarkers to detect abnormalities in the preliminary stages of pathology, well before clinical manifestation.

Sophisticated EEG techniques, when combined with other modalities, show promise in this regard. Combining EEG with event-related potentials could aid in memory evaluation, even in healthy individuals at risk for cognitive impairment. Machine learning algorithms utilizing EEG parameters have shown potential in predicting cognitive decline following epilepsy with high accuracy. Quantitative EEG has been associated with other AD markers and can distinguish between dementia types, making it valuable for prognosis, diagnosis, and monitoring [37]. Brain network changes in AD have predictive value for epilepsy, allowing for early intervention [5]. The concept of an “ictal network” in AD better describes the underlying epileptogenic mechanism than the term “epileptogenic zone.” [136]. Machine learning techniques applied to FDG-PET such as machine learning-based AD designation (MAD) and support vector machine iterative single data algorithm (SVM-ISDA) have been used to identify dementia risk in epileptic patients [137].

In a comprehensive study integrating CSF analysis, imaging, and neuropsychological assessments, LOEU patients exhibited lower MMSE scores accompanied by cognitive deficits across multiple domains. These findings were concomitant with CSF alterations indicative of AD pathology and reduced glucose metabolism in the right posterior cingulate cortex and left precuneus regions, reflecting neuronal dysfunction in these areas [138]. Nevertheless, a study integrating clinical, FDG-PET, and neuropsychological analyses compared LOEU to AD patients, revealing distinct patterns of abnormalities. This study concluded that AD may not be the primary cause of cognitive decline in LOEU, attributing cognitive impairment to the effects of seizures. Additionally, cerebrovascular disease was suggested to have a greater impact on cognitive decline compared to AD, based on the observed patterns of findings. These findings underscore the importance of identifying and treating vascular risk factors in patients with LOE [139]. Taken together, the combination of diverse biomarkers holds promise for researchers and clinicians in examining shared mechanisms, interactions, and treatment strategies on a case-by-case basis. However, further research and standardization efforts are needed to fully realize the potential of biomarkers in diagnosing and managing LOEU and its association with AD.

Epilepsy has a dual impact on cognition, exacerbating AD-related pathology and accelerating brain degeneration by inducing neuronal network alterations even before cognitive decline occurs [60]. Consequently, prompt identification and treatment of epileptic activity in AD, as well as screening of LOEU patients for AD biomarkers are crucial for mitigating or delaying cognitive decline. Newer anti-epileptic drugs offer several advantages in terms of cognitive outcomes and should be preferred over older medications. Levetiracetam, in particular, is undergoing extensive testing in this patient population due to its potential to reverse pathological changes and enhance cognition. Multiple mechanisms of action of levetiracetam are currently under investigation, with the results of clinical trials eagerly awaited. Hence, EEG biomarkers should be prioritized as they are currently applicable and could potentially guide treatment decisions, warranting careful elaboration for prompt integration into clinical practice. The significance of other discussed biomarkers will likely increase following the initial results of ongoing treatment studies.

Our review exhibits several strengths. Firstly, unlike previous reviews, it comprehensively addresses multiple domains implicated in the correlation between AD and epilepsy, reinforcing the concept of an epileptic variant of AD based on recent studies. Furthermore, beginning with an extensive analysis of the shared pathophysiological mechanisms between these two disorders, the necessity for more precise and timely diagnosis is emphasized. In this context, our review places a particular emphasis on the identification and discussion of novel biomarkers, highlighting their potential role in enhancing diagnostic accuracy and therapeutic interventions. Integrating this information with the clinical data, particularly concerning LOEU and SEA, enables clinicians to enhance patient outcomes by increasing vigilance and adopting precision medicine approaches. Finally, the findings presented herein may inform the development of prognostic, diagnostic, and therapeutic algorithms.

However, this review has several limitations. Primarily, numerous studies investigating the comorbidity between AD and epilepsy rely on retrospective data. In these studies, epilepsy is often reported generically, without analysis of specific subtypes. Additionally, a significant portion of the data is derived from animal models of AD and epilepsy, which may not accurately represent LOEU or sporadic AD, thus complicating the extrapolation of findings to human studies. We also identified a paucity of well-designed studies and standardized methodologies in this field, which impedes the generation of robust and reproducible results and presents a significant challenge in drawing meaningful conclusions from the existing literature. To address this issue, it is crucial to establish uniform criteria for patient selection, age cutoffs, and dosing of anti-epileptic drugs in future research endeavors. Additionally, clear guidelines for identifying epileptic activity, including subclinical epileptic activity on EEG, are needed to better characterize patients and assess the effects of interventions on both cognition and epileptic activity. By implementing consistent criteria and methodologies across studies, researchers can facilitate more accurate comparisons and enhance the reliability of findings in this field.

In conclusion, it is evident that AD and LOEU share complex and bidirectional relationships. The identification of SEA in AD patients and the vigilant cognitive monitoring of those with LOEU are crucial for early intervention and optimized patient care. Clarifying common pathophysiological mechanisms has illuminated potential targets for treatments that could alleviate both cognitive decline and epileptic symptoms. Biomarker evaluation holds promise in stratifying patient groups at risk, enabling more precise diagnoses and targeted therapies. This review highlights significant strides in understanding and managing the comorbidity of AD and epilepsy, emphasizing the urgent need for standardized methodologies and robust clinical studies. Addressing these challenges will pave the way for personalized medicine approaches that optimize outcomes and quality of life for patients affected by these interconnected neurological disorders.

## Figures and Tables

**Table 1:** Common risk factors for Alzheimer’s disease and epilepsy.

Genetic: Mutations of *PSEN1*, *PSEN2*, *APP* and duplication of *APP* [28] *TREM2*—*R47H* variant [35], *APOEε4* allele [13] Other genetic loci [36]
Age [7,37]
Mild cognitive impairment [37]
Vascular [7]: Cerebrovascular factors [27] Cardiovascular risk factors [37,38]
History of brain traumatic injury [27]
Blood–brain barrier dysfunction [27]

*PSEN1*: presenilin 1; *PSEN2*: presenilin 2; *APP*: amyloid precursor protein; *TREM2*: Triggering receptor, expressed on myeloid cells 2; *APOE*: apolipoprotein E.

## Abbreviations

AD, Alzheimer disease; MCI, mild cognitive impairment; LOE, late-onset epilepsy; LOEU, late-onset epilepsy of unknown etiology; SEA, subclinical epileptiform activity; EEG, electroencephalograph; MEG, magnetoencephalography; aMCI, amnestic MCI; *PSEN1*, presenilin 1; *PSEN2*, presenilin 2; *APP*, amyloid precursor protein; IEDs, interictal epileptiform discharges; ACE, Addenbrooke’s cognitive examination; VLOM, Verbal-Language/Orientation-Memory; CSF, cerebrospinal fluid tTau, total tau protein; Aβ, amyloid-beta; MMSE, Mini-Mental State Examination; TREM2, Triggering receptor expressed on myeloid cells 2; *APOEε4*, apolipoprotein E gene ε4 allele; BBB, blood–brain barrier; pTau, hyperphosphorylated tau; GSK3β, Glycogen synthase kinase-3 beta; TLE, temporal lobe epilepsy; CDK5, cyclin-dependent kinase 5 PSD95, postsynaptic density protein 95; CNS, central nervous system; TNF-α, tumor necrosis factor-α; IL, interleukin; TLR, Toll-like receptor; NLRP3, leucine-rich repeat Pyrin Domain Containing 3; BACE1, Beta-secretase 1; mTOR, Mechanistic target of rapamycin; REM, rapid eye movement; FDZ7, Frizzled Class Receptor 7; TIRDA, temporal intermittent rhythmic delta activity; LTM-VEEG, long-term monitoring with video-EEG; M/EEG, magnetoencephalography with simultaneous EEG; Hd, high-density; Nfl, neurofilament light chain; FDG, Fluorodeoxyglucose; PET, positron emission tomography; MRI, Magnetic resonance imaging; fMRI, Functional MRI; SV2A, synaptic vesicle glycoprotein 2A; LEV, levetiracetam; BRV, brivaracetam; LAM, Lamotrigine; TOP, topiramate; LAC, Lacosamide; JNK, c-Jun N-terminal kinase; MAD, machine learning-based AD designation; SVM-ISDA, support vector machine iterative single data algorithm.
